# Their Keeping Properties

**Published:** 1920-10-16

**Authors:** 


### DRIED FRUIT AND VEGETABLES.

### Their Keeping Properties.

Lord Lee of Fareham, the Minister of Agriculture, in a speech made a short time ago to a mass meeting of farmers at Reading, stated that the world's food supply is in danger, and that there is a grave risk, not only of a shortage but of actual famine conditions.

This speech was made to food producers, and it is to be hoped that it will have its due effect. But with the problem of food production other problems go hand in hand, and protection of the food from spoilage during transport and storage is one of the most important of these. In America it has been computed that, as a result of bad crop management, bad market conditions, defective transport facilities, and kindred agencies, no less than 50 per cent. of the total food produced fails to reach the consumer. The importance, then, of any means of preserving the food without impairing either its nutritive value or flavour is immense.

Drying is an old and well-tried method of preserving both flesh, fruit, and vegetables, and a short account of some investigations undertaken in America to find out what conditions affected dried fruit and vegetables, favourably and unfavourably, both during preparation and storage may prove of value.

The dried foods on the market contain less than 10 per cent. of water; they have frequently been treated with hot water or steam, and have been washed. The actual drying takes place at a temperature of from 130° to 185° F., and lasts for from 3 to 24 hours. Products so treated should, when soaked in water, reabsorb the water previously removed, and reassume their normal appearance.

The results of cultural experiments were as follows:—Few samples of dried vegetables were found to be sterile, though there were often very few bacteria: when suitably kept these organisms tended to decrease; the spores of moulds were constantly found; yeasts were never found. For example, a specimen of dried peaches gave the following results:—

Water 2.79 per cent., total bacteria per gram 800, total moulds per gram 40, which results compare very favourably with those obtained from the fresh fruit, which is very satisfactory.

Experiments on storage were conducted as follows:—The fruits or vegetables were packed in sealed tins, in glass jars, and in waxed cardboard containers, and these were stored in (1) a temperature of 98° F. in a very damp atmosphere, (2) at 65° F. in a slightly damp atmosphere, (3) at room temperature and at the normal degree of humidity, and (4) at freezing temperature in the damp. Though in sealed tins, jars, or cartons, no attempt was made to sterilise the fruit.

The results were as follows: When packed in sealed tins or glass jars, no matter how stored, the amount of moisture and the number of mould spores remained constant. The number of bacteria decreased with the time.

When packed in waxed cardboard cartons and stored in warm (98° F.) damp places there was no change for the first two weeks; in the next fortnight the amount of water in the sample was doubled, and in another fourteen days the increase of water was anything from 400 to 1,000 per cent., from which it seems that waxed paper is pervious to water vapour, though not to water in liquid form. With the increase in the water, growth either of bacteria or moulds, or both took place, and the food was spoiled. Cardboard containers stored in the damp at freezing point showed a similar increase in water content, and were spoiled by bacterial growth in many cases. Samples in cardboard at normal room temperature and humidity remained perfectly good, the water content remaining normal, the bacteria decreasing, and the moulds remaining constant in character.

The investigator concludes that dried fruit and vegetables packed in waxed paper cartons are suitable for use in temperate climates only, while tins or jars will be useful anywhere. That as to the risk of poisoning it is little if any greater than with fresh fruit, though it is recognised that there is a distinct danger in eating such products uncooked. As to nutritional value, it seems to be unimpaired.

